# Cardiac Myosin Binding Protein C and MAP-Kinase Activating Death Domain-Containing Gene Polymorphisms and Diastolic Heart Failure

**DOI:** 10.1371/journal.pone.0035242

**Published:** 2012-04-17

**Authors:** Cho-Kai Wu, Yin-Tsen Huang, Jen-Kuang Lee, Liang-Ting Chiang, Fu-Tien Chiang, Shu-Wei Huang, Jiunn-Lee Lin, Chuen-Den Tseng, Yau-Hung Chen, Chia-Ti Tsai

**Affiliations:** 1 Division of Cardiology, Department of Internal Medicine, National Taiwan University College of Medicine and Hospital, Taipei, Taiwan; 2 Graduate Institute of Clinical Medicine, College of Medicine, National Taiwan University, Taipei, Taiwan; 3 Department of Family Medicine and Health Management Center, Far Eastern Memorial Hospital, New Taipei City, Taiwan; 4 Department of Laboratory Medicine, National Taiwan University Hospital, Taipei, Taiwan; 5 Department of Clinical Pathology, Far Eastern Memorial Hospital, New Taipei City, Taiwan; 6 Graduate Institute of Biomedical Electronics and Bioinformatics, National Taiwan University, Taipei, Taiwan; 7 Graduate Institute of Biomedical Engineering, College of Medicine, National Yang-Ming University, Taipei, Taiwan; 8 Department of Chemistry, Tamkang University, Tamsui, Taiwan; National Taiwan University Hospital, Taiwan

## Abstract

**Objective:**

Myosin binding protein C (MYBPC3) plays a role in ventricular relaxation. The aim of the study was to investigate the association between *cardiac myosin binding protein C* (*MYBPC3*) gene polymorphisms and diastolic heart failure (DHF) in a human case-control study.

**Methods:**

A total of 352 participants of 1752 consecutive patients from the National Taiwan University Hospital and its affiliated hospital were enrolled. 176 patients diagnosed with DHF confirmed by echocardiography were recruited. Controls were matched 1-to-1 by age, sex, hypertension, diabetes, renal function and medication use. We genotyped 12 single nucleotide polymorphisms (SNPs) according to HapMap Han Chinese Beijing databank across a 40 kb genetic region containing the *MYBPC3* gene and the neighboring DNA sequences to capture 100% of haplotype variance in all SNPs with minor allele frequencies ≧5%. We also analyzed associations of these tagging SNPs and haplotypes with DHF and linkage disequilibrium (LD) structure of the *MYBPC3* gene.

**Results:**

In a single locus analysis, SNP rs2290149 was associated with DHF (allele-specific *p* = 0.004; permuted *p* = 0.031). The SNP with a minor allele frequency of 9.4%, had an odds ratio 2.14 (95% CI 1.25–3.66; *p* = 0.004) for the additive model and 2.06 for the autosomal dominant model (GG+GA : AA, 95% CI 1.17–3.63; *p* = 0.013), corresponding to a population attributable risk fraction of 12.02%. The haplotypes in a LD block of rs2290149 (C-C-G-C) was also significantly associated with DHF (odds ratio 2.10 (1.53–2.89); permuted p = 0.029).

**Conclusions:**

We identified a SNP (rs2290149) among the tagging SNP set that was significantly associated with early DHF in a Chinese population.

## Introduction

Heart failure is a common hemodynamic and neurohormonal syndrome, the prevalence of which is increasing as the population ages [1]. Diastolic heart failure (DHF) and systolic heart failure (SHF) are the two most commonly encountered forms of heart failure in clinical practice. Clinically, overt DHF and SHF appear to be two separate syndromes that have distinctive morphologic and functional change characteristics; however the signs, symptoms, and prognoses of both syndromes are very similar [2]. Structurally, patients with SHF have eccentric left ventricular (LV) hypertrophy, whereas patients with DHF have concentric LV hypertrophy [3]. From a molecular perspective, the cytoskeletal protein titin, matrix metalloproteinase degradation, and impaired calcium homeostasis have been linked with DHF [4]–[7]. We have previously reported on genetic factors that may contribute to the development and prognosis of DHF [8], [9]. However, the precise genetic etiology of DHF remains poorly understood.

The *Myosin binding protein C* (*MYBPC3*) gene encodes myosin binding protein C, a key constituent of the thick filaments localized to doublets in the C-zone of the A-band of the sarcomere. By binding to myosin, titin and actin, MYBPC3 contributes to maintaining the structural integrity of the sarcomere and regulates cardiac contractility in response to adrenergic stimulation [10]. Findings from a recent study suggest that MYBPC3 can inhibit the myosin-actin interaction, allowing complete relaxation during diastole, and that MYBPC3 ablation causes defective diastolic relaxation [11]. *MYBPC3* gene mutations have also been demonstrated to be associated with a risk of delayed, mild hypertrophic heart failure [12] and hypertrophic cardiomyopathy (HCM) [13]. However, findings from a mouse gene knock-out study have revealed that *MYBPC3* gene mutation was associated with concentric LV hypertrophy and mildly impaired contractile function, a characteristic feature of DHF [14] Given these findings we hypothesized that *MYBPC3* gene variations may affect an individual's susceptibility to develop DHF.

Recently a 25-bp deletion polymorphism in intron 32 of the *MYBPC3* gene was identified in south Asians and found to be associated with an increased risk of cardiomyopathy [12]. In the present study, we investigated if this common deletion variant was also present in a Han Chinese population and if the variant was associated with an increased risk of DHF. We also screened for other common *MYBPC3* variants in the same Han Chinese population. Single nucleotide polymorphisms (SNPs) tagging common variations across the *MYBPC3* gene were selected according the HapMap Han Chinese Beijing databank (CHB) group (http://www.hapmap.org/). Associations between DHF and linkage disequilibrium (LD) structure among the *MYBPC3* gene tagging SNP set were analyzed. We found that there was no deletion variant in our population. However, we found for the first time that a SNP among the tagging SNP set was significantly associated with an increased risk of early DHF.

## Methods

### Ethics Statement

We certify that all the applicable institutional and governmental regulations concerning the ethical use of human volunteers/animals were followed during this research. Written informed consent was obtained from every participating subject, and the study was approved by the institutional review board of the National Taiwan University Hospital (approval ID: 20070313R).

### Screening for the *MYBPC3* gene deletion

A 25 bp deletion in intron 32 of *MYBPC3* gene was previously reported to be associated with cardiomyopathies, including hypertrophic and dilated cardiomyopathies. We screened DNA samples from 400 individuals in a population of Taiwanese patients with cardiomyopathies. The methods used have been described in detail previously [12].

### Case-control study participants and study design

A total of 1752 consecutive patients from the cardiovascular ward or clinic of the National Taiwan University Hospital and an affiliated hospital were recruited from July 2005 through April 2008. All patients underwent echocardiography. Diastolic heart failure was defined as previously described and according to recent guidelines [8], [15]. Briefly, the definitions used included: exertional dyspnea (New York Heart Association function class II–III); heart failure as defined by the Framingham criteria and normal systolic function (ejection fraction ≥50%); and echocardiographic evidence of LV diastolic dysfunction ie, a mitral inflow E/A ratio <1, deceleration time >220 cm/s, and decreased peak annular early diastolic velocity of the lateral mitral annulus <8 cm/s upon tissue Doppler imaging. Patients who had renal failure, significant hepatic disease, secondary hypertension, a history of myocardial infarction, significant coronary artery disease (CAD; coronary artery stenosis ≥70%), pericardial disease, significant valvular heart disease (≥moderate), chronic obstructive pulmonary disease, or chronic atrial fibrillation were excluded. The control population comprised risk-factor matched control patients. For every case patient, a matched control with no symptoms of heart failure and no objective evidence of diastolic dysfunction (a mitral inflow E/A ratio between 1∼2, deceleration time <220 cm/s, and decreased peak annular early diastolic velocity of the lateral mitral annulus >8 cm/s upon tissue Doppler imaging) was selected from the same ward or clinic. Cases and controls were individually matched by sex, age (difference≤5 years), blood pressure, diabetes status, renal function, and medication use. A total of 176 patients with DHF (96 men and 80 women) and 176 matched controls were selected for study.

### Echocardiography

Left atrial diameter, LV end diastolic and systolic diameter, interventricular septum thickness, LV posterior wall thickness, mitral inflow early rapid filling wave (E), peak velocity of the late filling wave due to atrial contraction (A), the E/A ratio, E wave deceleration time, and mitral annular early diastolic velocity were measured according to American Society of Echocardiography guidelines using a Sonos 7500 echocardiography probe (Philips, Andover, MA) attached to a 1 to 3 MHz transducer. The LV ejection fraction was calculated as previously described [12]. The LV mass index (LVMI) was calculated from the LV end diastolic and systolic diameter, interventricular septum thickness, and LV posterior wall thickness according to the method of Devereux et al [14]. Doppler and color Doppler studies were performed to detect valvular heart disease. Significant valvular heart disease was defined as at least moderate aortic or mitral stenosis/regurgitation.

### Selection of SNPs and genotyping

Using the HapMap CHB databank (public data release 21 a/phase II, Jan. 2007), 103 SNPs were identified in a 40 kb gene region containing the *MYBPC3* gene and parts of an upstream (*SPI1*) and downstream gene (*MADD*). To identify common haplotype tagging SNPs, eligible SNPs were entered into the Tagger program in Haploview version 3.32 [16]. (http://www.broad.mit.edu/mpg/haploview/). The minor allele frequency threshold was 5% (*r* ^2^ = 0.8. Twelve tag SNPs (rs10838692, rs11039179, rs2290149, rs7124958, rs753992, rs2305280, rs10769253, rs2856650, rs2697920, rs1057233, rs3824869, and rs3740689) were selected, capturing 100% of haplotype variance for all SNPs on the *MYBPC3* gene with minor allele frequencies ≥5%. All SNP markers were genotyped by matrix-assisted laser desorption/ionization-time of flight mass spectrometry (MALDI-TOF MS) [15], [17]. A DNA fragment (100–300 bp) encompassing the SNP site was amplified using a polymerase chain reaction GeneAmp 9700 thermocycler (Applied Biosystems, Foster City, CA) according to the manufacturer's instructions. After polymerase chain reaction amplification and neutralization of the deoxynucleotide triphosphates (dNTP), primer extension was performed by adding the probe, Thermo Sequenase (Amersham Pharmacia, Piscataway, NJ), and an appropriate dideoxynucleotide triphosphate/dNTP mixture. Extension products were differentiated by MALDI-TOF. We then compared the reference allele frequency and MAF of our population with other populations announced in the HapMap databank (Tables S1).

### Statistical analysis

Baseline characteristics and echocardiographic findings were compared between groups using Student's unpaired *t*-test (continuous data) or chi-square test (categorical data). We calculated the power of the single locus analyses using the Genetic Power Calculator (http://pngu.mgh.harvard.edu/~purcell/gpc/) [18]. To detect an odds ratio of 1.6 (autosomal dominance), with a two-sided alpha error of 0.05, the calculated power was 0.57 to 0.90, with the percentage of having at least one variant allele (dominant model) ranging from 0.1 to 0.5. The association between each SNP and haplotype and DHF was estimated using Haploview software. A Hardy-Weinberg equilibrium (HWE) test was performed for each sequence variant of the control group before marker-trait association analysis [16]. Nominal two-sided *P* values were corrected for multiple tests by 10,000 times permutation. For haplotype construction, genotype data from the case and control groups were used to estimate intermarker linkage disequilibrium (LD) by measuring pairwise D′ and *r* ^2^ and defining LD blocks. We used the confidence interval (CI) method component of the Haploview software to define an LD block with an extended spine if D′ was >0.8 [17]. The population attributable risk (PAF) was estimated data from the control group data as follows: 1−{1/[*p*2 OR_homo_+2*p*(1−*p*)OR_hetero_+(1−*p*)^2^]}, where *p* is the risk-allele frequency, OR_homo_ is the odds ratio (OR) for homozygotes, and OR_hetero_ is the OR for heterozygotes.

## Results

### Baseline characteristics

The baseline characteristics of the study participants are shown in Table 1. All baseline characteristics were similar between the case and control groups. The echocardiographic parameters of the study participants are shown in Table 2. Although IVS and LVPW thicknesses were higher in the DHF group, there was no between group difference in LVMI. This suggests that the degree of LV hypertrophy, which can affect diastolic function, was similar between the groups. Case group participants had a significantly lower mitral inflow E/A ratio, a longer mitral inflow deceleration time, and a shorter mitral annular early diastolic velocity than control group participants. The participants of our current study are similar to the recent prospective, multi-centered, randomized, open-label study in Chinese DHF patients [19]. Most participants in the DHF group were classified as having at least grade I diastolic dysfunction (Table 2) [20].

**Table 1:** Baseline characteristics of patients and controls.

Characteristic	DHF (n = 176)	Matched Controls (n = 176)	*P*
Age (years)	63.7±10.9	62.3±11.1	0.23
Sex (male/female)	96/80	96/80	1.00
BMI (kg/m^2^)	25.6±3.2	24.2±2.9	<0.001
Diabetes mellitus (%)	13	10	0.13
Hypertension (%)	59	58	0.91
Creatinine (mg/dL)	0.99±0.28	0.98±0.23	0.8
Anti-hypertension therapy			
ACEI+ARB	26	20	0.31
β-blocker	34	30	0.49
Calcium channel blocker	49	41	0.11

DHF, diastolic heart failure; BMI, body mass index; BP, blood pressure; ACEI, angiotensin-converting enzyme inhibitor; ARB, angiotensin II type I receptor blocker.

**Table 2:** Echocardiographic data.

Variable	DHF (n = 176)	Control (n = 176)	*P*
LA diameter (mm)	35.0±5.1	34.2±5.8	0.21
LVEF (%)	70.5±7.9	71.3±8.0	0.35
IVS (mm)	11.6±1.8	10.9±1.7	<0.001
LVPW (mm)	11.1±1.9	10.5±1.6	0.003
LVEDD (mm)	45.1±5.1	45.4±5.4	0.56
LVESD (mm)	26.8±5.2	26.8±5.2	0.93
LVMI (g/m^2^)	126.67±37.23	116.59±36.84	0.15
E (cm)	62.9±15.9	84.3±19.3	<0.001
A (cm)	85.9±15.8	67.9±18.5	<0.001
DT (cm/s)	242.8±41.9	162.8±40.1	<0.001
Mitral Ea (cm/s)	7.3±1.9	10.9±2.05	<0.001

DHF, diastolic heart failure; LA, left atrium; LVEF, left ventricular ejection fraction; IVS, interventricular septum; LVPW, left ventricular posterior wall; LVEDD, left ventricular end diastolic dimension; LVESD, left ventricular end systolic dimension; LVMI, left ventricular mass index; E, mitral valve ejection flow; A, mitral valve atrium flow; DT, deceleration time; Mitral Ea, peak mitral annular early diastolic velocity.

### *MYBPC3* gene deletion

We screened 400 healthy Taiwanese individuals for the 25 bp *MYBPC3* gene deletion polymorphism. None of the screened individuals had the previously described [12] 25 bp gene deletion in intron 32 of the *MYBPC3* gene.

### Characteristics of the SNPs and LD structures of *MYBPC3* gene

**Figure 1:**
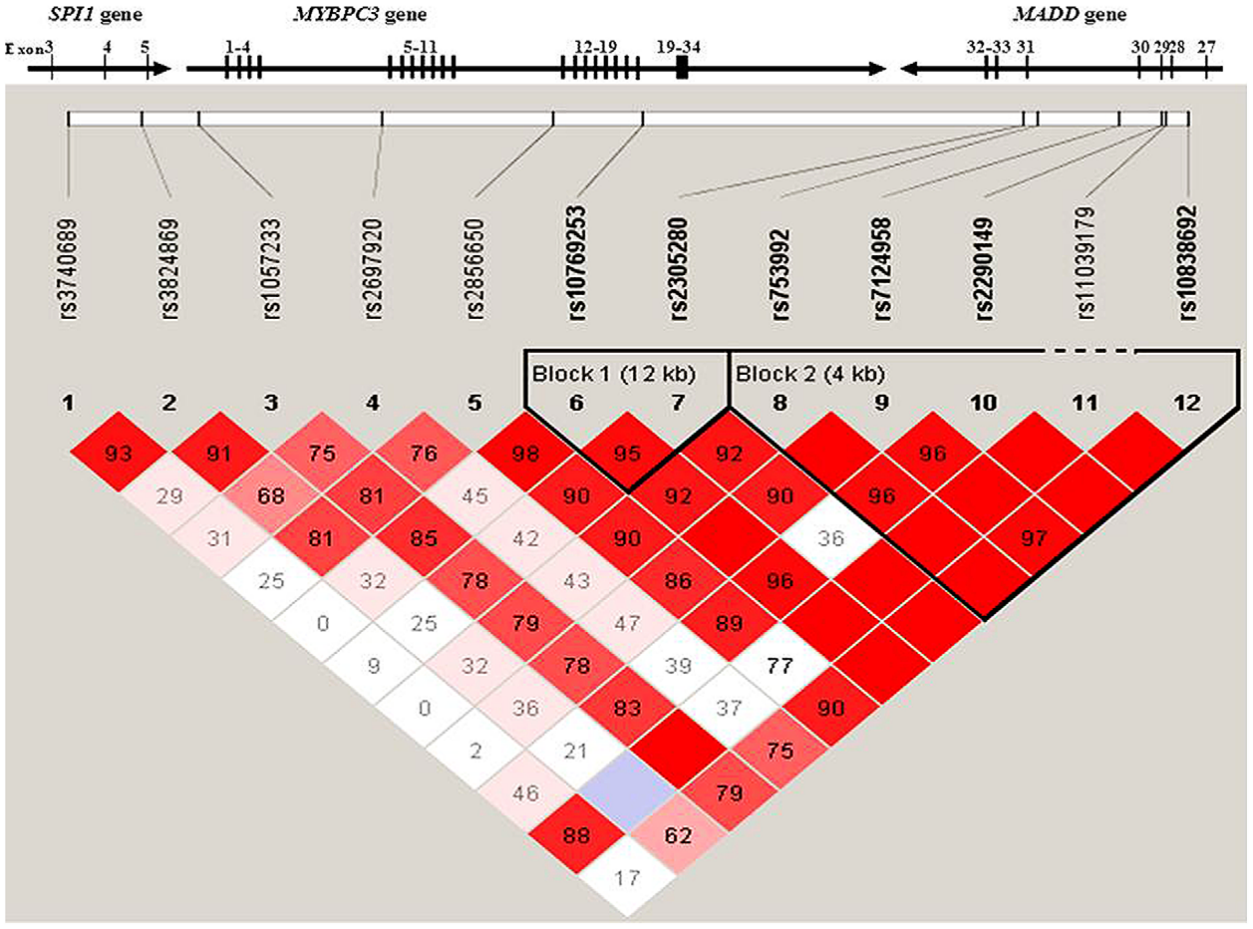
Graphical representation of SNPs in relation to the exon-intron structure (upper line) and Haploview LD graph of the *MYBPC3* gene and the adjacent *SPI1* and *MADD* genes (lower panel). The exon regions are shown with filled rectangles and are numbered in order. Pairwise LD coefficients D′×100 are shown in each cell (D′ values of 1.0 are not shown). A standard Haploview color scheme was applied for the LD color display (logarithm of odds [LOD] score ≥2 and D′ = 1, shown in bright red; LOD score ≥2 and D′<1 shown in pink; LOD score<2 and D′ = 1 shown in blue; LOD score<2 and D′<1 shown in white).

On average, 99.15% of attempted genotypes were successful (success rates ranged from 97.8% to 99.9% for each SNP). The concordance rate of genotyping duplication was 99.37%. The *MYBPC3* gene, located on chromosome 11, is 22 kb in length with 34 exons and introns. The adjacent genes are *SPI1* (upstream) and *MADD* (downstream). A graphic representation of the SNPs in relation to the exon-intron structure (according to the National Center for Biotechnology Information) is shown in Figure 1. Most SNPs were located in introns, except for SNP rs1057233, which was located in the 5′ upstream promoter region. The genomic position, nucleic acid composition, HWE test *P* values, and minor allele frequencies of the 12 genotyped SNPs are summarized in Table 3.

**Table 3:** Association between *MYBPC3* and *MADD* gene sequence variants and Diastolic Heart Failure.

No.	SNP name	Gene position (kb)	Gene region	Minor/major allele	MAF		HW *P* value	OR (95% CI)	Allelic *P*	Permuted *P* *
					Case	Control				
1	rs3740689	−6.41	*SPI1*geneIntron 3	A/G	0.463	0.423	0.236	1.19 (0.88–1.60)	0.288	0.890
2	rs3824869	−4.07	*SPI1* geneIntron 4	T/C	0.293	0.273	0.028	1.10 (0.79–1.53)	0.558	0.99
3	rs1057233	−2.27	5′ Upstreampromoter	C/T	0.259	0.304	0.023	0.80 (0.57–1.11)	0.180	0.759
4	rs2697920	3.56	Intron 5	A/G	0.463	0.480	0.616	0.93 (0.69–1.26)	0.651	0.99
5	rs2856650	8.97	Intron 11	T/C	0.276	0.324	0.446	0.79 (0.57–1.10)	0.162	0.724
6	rs10769253	11.83	Intron 19	G/A	0.426	0.412	0.858	1.06 (0.79–1.43)	0.702	0.99
7	rs2305280	23.88	*MADD* gene Intron 32	C/T	0.398	0.372	0.435	1.11 (0.89–1.51)	0.486	0.989
9	rs753992	24.32	*MADD* gene Intron 30	C/T	0.420	0.418	0.403	1.01 (0.75–1.36)	0.939	1.00
9	rs7124958	26.91	*MADD* gene Intron 30	C/T	0.406	0.381	0.484	0.90 (0.66–1.22)	0.488	0.99
10	rs2290149	28.25	MADD gene Intron 29	G/A	0.125	0.062	0.728	2.14 (1.25–3.66)	0.004	0.031
11	rs11039179	28.37	*MADD* gene Intron 28	T/C	0.034	0.031	0.620	1.09 (0.48–2.51)	0.832	1.00
12	rs10838692	29.07	*MADD* gene Intron 27	T/C	0.287	0.321	0.304	0.85 (0.62–1.18)	0.326	0.948

UTR, untranslated region; HW, Hardy-Weinberg; MAF, minor allele frequency.

* Permutation 10,000 times.

### *MYBPC3* gene polymorphisms and DHF association analysis

To determine the extent of LD in the *MYBPC3* gene, genotype data from both the case and control groups were used to estimate intermarker LD. Standardized pairwise LD coefficients D′ and *r* ^2^ between markers were estimated and two LD blocks were identified across the gene (Figure 1). We found that rs2290149 was significantly associated with DHF (nominal *P* = 0.004) (Table 3). The association remained significant after correcting for multiple testing (permuted *P* = 0.031) (Table 3). The SNP with a minor allele frequency of 9.4%, located 28.25 kb downstream of the gene origin and in the 29^th^ intron of the *MADD* gene, was significantly associated with DHF for both dominant models (OR 2.06 [95% CI 1.17–3.63, *P* = 0.013]) and had a PAF of 12.02%. This SNP was located in a LD block (LD block 2) spanning intron 27 to intron 32 of *MADD* gene (Figure 1). The haplotypes within this LD block were significantly associated with DHF. Among these haplotypes, the haplotype C-C-G-C was associated with an increased risk (OR 2.10 [95% CI 1.53–2.89], permuted *P* = 0.029) of DHF (Table 4). These findings suggest that there are strong associations between LD block 2 and DHF.

**Table 4:** *MYBPC3* and *MADD* gene haplotype/DHF association analysis.

Haplotype	Frequencies		OR (95% CI)	Nominal *P*	Permuted *P* *
	Case	Control			
Block 1					
A-T	0.556	0.585	0.89 (0.74–1.06)	0.4466	1.000
G-C	0.380	0.369	1.05 (0.87–1.26)	0.7552	1.000
G-T	0.046	0.043	1.07 (0.70–1.64)	0.8556	1.000
A-C	0.017	0.003	5.75 (1.68–19.68)	0.063	0.2458
Block 2					
T-T-A-C	0.577	0.582	0.98 (0.82–1.17)	0.8786	1.000
C-C-A-T	0.281	0.318	0.84 (0.69–1.02)	0.2849	0.8334
C-C-G-C	0.122	0.062	2.10 (1.53–2.89)	0.0063	0.0295
C-T-A-C	0.009	0.034	0.26 (0.12–0.54)	0.0188	0.0884

* Permutation 10,000 times.

## Discussion

The present study shows an association between a specific SNP and early DHF independent of hypertension. We used a pure Han-Chinese population to eliminate false-positive results due to population stratification. The current results demonstrated a novel SNP that may predispose to development of DHF in the Han-Chinese population.

The *MYBPC3* gene encodes myosin-binding protein, a large multidomain protein which comprises 11 modules, C0 to C10 (N terminus to C terminus). The protein has been reported to play important roles in both health and disease [21]. The MYBPC3 isoform contains protein kinase A phosphorylation sites within the S2 binding site that are absent in skeletal muscle isoforms. Further, recent data suggest that phosphorylated MYBPC3 may be cardioprotective [22]. Variable disease phenotypes and prognoses have been reported with different *MYBPC3* gene mutations. However, the findings from most studies suggest that *MYBPC3* mutations are associated with late-onset, mild hypertrophy and incomplete penetrance [23], [24], as per DHF.

Recently, Dhandapany and colleagues reported that individuals in a south Asian population who were heterozygous for a common *MYBPC3* variant (a 25 bp deletion) had an increased risk of late onset, mild HCM [12]. We found that there was no such deletion in a homogenous Han Chinese population. We systemically screened the tagSNP from the upstream promoter to the downstream neighboring gene. We studied a homogenous Taiwanese population to eliminate false-positives due to population stratification. Further, patients with CAD were excluded from our study and the percentage of patients with hypertension was nearly identical in both the DHF group and the control group. Therefore, the confounding effects of CAD and hypertension (the most common risk factors for DHF) were eliminated. In our cohort, DHF was caused by diastolic dysfunction, or more specifically, by abnormalities in active relaxation or passive stiffness of the LV.

To elucidate the mechanism by which MYBPC3 causes disease and structural changes, knockout mice, heterozygous (+/−) and homozygous (−/−), were produced by deleting exons 3 through 10 on the endogenous cardiac *MYBPC3* gene [14]. Studies have revealed that MYBPC3 +/− mice are indistinguishable from wild-type control mice whereas MYBPC3 −/− mice exhibit significant cardiac hypertrophy. Removal of MYBPC3 can increase the velocity of shortening and force output [25] and lower the amount of phosphorylated MYBPC3 in the failing human heart [26]. We suspect that a minor modification to the *MYBPC3* gene, such as a SNP or DNA phosphorylation, might contribute to the development of HCM. Interestingly, the SNP rs2290149 identified in our study to be associated with DHF was located in an LD block containing the 3′ untranslated region of the *MYBPC3* gene and the *MADD* gene. Whether this SNP plays a role in regulating *MYBPC3* gene expression or is in LD with another functional polymorphism of the *MYBPC3* gene remains to be determined. Our findings suggest that the *MADD* gene may also be associated with an increased risk of DHF.

The *MADD* gene encodes MAPK-activating death domain-containing protein. This protein can modulate the tumor necrosis factor-alpha (TNF-α) activated NF-kappa beta, MAPK, ERK1/2, JNK, and p38 pathway by interacting with TNFR1. In vitro studies have demonstrated that *MADD* plays an essential in counteracting the actions of TNF-α [27]. Tumor necrosis factor-alpha has been found to induce time-dependent phosphorylation of ERK, MSK1, and phospholamban on the threonine 17 residue via TNFR1 [7]. Low concentrations of TNF-α (200 to 500 U/mL) acutely decrease action potential duration, peak Ca^2+^ transient amplitude, and the rate of Ca^2+^ decline [28]. In addition, TNF-α can also mediate β-AR sensitivity by nitrous oxide-dependent mechanisms, thus leading to diastolic dysfunction [29]. Further in vitro and gene mapping studies are needed to determine whether the SNPs in the *MADD* gene are associated with altered activation of the aforementioned pathways warrants. The SNP rs2290149 is located in a genetic cluster of MYBPC3 and MADD gene. The haplotype that associated with DHF contained the regions of both genes. Therefore, both of the above genes are possible targets to be associated with DHF. Further fine mapping and functional studies are warranted to prove whether *MADD* gene are associated with DHF or that the SNP is involved in the regulation of *MYBPC3* gene. Our current study could only provide evidence that tag SNP rs2290149 is associated with the development of DHF.

Our study has several limitations that warrant mention. Of note, our sample size was relatively small. However, we used an individual risk-factor matched design to select control patients, substantially increasing statistical efficiency and power and decreasing the number of case-control pairs needed to obtain a significant result. Further studies of other ethnic populations are needed. According to our current definition, patients with moderate and severe DHF were not included in this study and the results should not be extrapolated to patients with moderate or severe DHF.

In conclusion, we suggest that development of DHF may have a genetic component. We identified the genetic variant SNP rs2290149 to be associated with DHF and have a substantial PAF in the Chinese population studied.

## Supporting Information

Table S1 **Comparison of** ***MYBPC3*** **and** ***MADD*** **gene sequence variants in our population, Han Chinese, Yoruba in Ibadan, Nigeria Japanese in Tokyo, Japan and CEPH (Utah residents with ancestry from northern and western Europe).** MAF, minor allele frequency; YRI, Yoruba in Ibadan, Nigeria; JPT, Japanese in Tokyo, Japan; CHB, Han Chinese in Beijing, China; CEU, CEPH (Utah residents with ancestry from northern and western Europe).
(DOCX)

## References

[1] Cowie MR, Mosterd A, Wood DA, Deckers JW, Poole-Wilson PA (1997). The epidemiology of heart failure.. Eur Heart J.

[2] Chatterjee K, Massie B (2007). Systolic and diastolic heart failure: Differences and similarities.. J Card Fail.

[3] Katz AM, Zile MR (2006). New molecular mechanism in diastolic heart failure.. Circulation.

[4] Borbely A, Falcao-Pires I, van Heerebeek L, Hamdani N, Edes I (2009). Hypophosphorylation of the stiff n2b titin isoform raises cardiomyocyte resting tension in failing human myocardium.. Circ Res.

[5] Spinale FG, Coker ML, Heung LJ, Bond BR, Gunasinghe HR (2000). A matrix metalloproteinase induction/activation system exists in the human left ventricular myocardium and is upregulated in heart failure.. Circulation.

[6] Leszek P, Szperl M, Klisiewicz A, Janas J, Rozanski J (2008). Alterations in calcium regulatory protein expression in patients with preserved left ventricle systolic function and mitral valve stenosis.. J Card Fail.

[7] Defer N, Azroyan A, Pecker F, Pavoine C (2007). Tnfr1 and tnfr2 signaling interplay in cardiac myocytes.. J Biol Chem.

[8] Wu CK, Luo JL, Wu XM, Tsai CT, Lin JW (2009). A propensity score-based case-control study of renin-angiotensin system gene polymorphisms and diastolic heart failure.. Atherosclerosis.

[9] Wu CK, Luo JL, Tsai CT, Huang YT, Cheng CL (2010). Demonstrating the pharmacogenetic effects of angiotensin-converting enzyme inhibitors on long-term prognosis of diastolic heart failure.. Pharmacogenomics J.

[10] McClellan G, Kulikovskaya I, Winegrad S (2001). Changes in cardiac contractility related to calcium-mediated changes in phosphorylation of myosin-binding protein c.. Biophys J.

[11] Pohlmann L, Kroger I, Vignier N, Schlossarek S, Kramer E (2007). Cardiac myosin-binding protein C is required for complete relaxation in intact myocytes.. Circ Res.

[12] Dhandapany PS, Sadayappan S, Xue Y, Powell GT, Rani DS (2009). A common mybpc3 (cardiac myosin binding protein c) variant associated with cardiomyopathies in south asia.. Nat Genet.

[13] Girolami F, Ho CY, Semsarian C, Baldi M, Will ML (2010). Clinical features and outcome of hypertrophic cardiomyopathy associated with triple sarcomere protein gene mutations.. J Am Coll Cardiol.

[14] Harris SP, Bartley CR, Hacker TA, McDonald KS, Douglas PS (2002). Hypertrophic cardiomyopathy in cardiac myosin binding protein-c knockout mice.. Circ Res.

[15] Wu CK, Tsai CT, Chang YC, Luo JL, Wang YC (2009). Genetic polymorphisms of the angiotensin ii type 1 receptor gene and diastolic heart failure.. J Hypertens.

[16] Barrett JC, Fry B, Maller J, Daly MJ (2005). Haploview: Analysis and visualization of ld and haplotype maps.. Bioinformatics.

[17] Jurinke C, van den Boom D, Cantor CR, Koster H (2002). The use of massarray technology for high throughput genotyping.. Adv Biochem Eng Biotechnol.

[18] Purcell S, Cherny SS, Sham PC (2003). Genetic power calculator: Design of linkage and association genetic mapping studies of complex traits.. Bioinformatics.

[19] Yip GW, Wang M, Wang T, Chan S, Fung JW (2008). The Hong Kong diastolic heart failure study: a randomised controlled trial of diuretics, irbesartan and ramipril on quality of life, exercise capacity, left ventricular global and regional function in heart failure with a normal ejection fraction.. Heart.

[20] Angeja BG, Grossman W (2003). Evaluation and management of diastolic heart failure.. Circulation.

[21] Flashman E, Redwood C, Moolman-Smook J, Watkins H (2004). Cardiac myosin binding protein c: Its role in physiology and disease.. Circ Res.

[22] Sadayappan S, Osinska H, Klevitsky R, Lorenz JN, Sargent M (2006). Cardiac myosin binding protein c phosphorylation is cardioprotective.. Proc Natl Acad Sci U S A.

[23] Niimura H, Bachinski LL, Sangwatanaroj S, Watkins H, Chudley AE (1998). Mutations in the gene for cardiac myosin-binding protein c and late-onset familial hypertrophic cardiomyopathy.. N Engl J Med.

[24] Kubo T, Kitaoka H, Okawa M, Matsumura Y, Hitomi N (2005). Lifelong left ventricular remodeling of hypertrophic cardiomyopathy caused by a founder frameshift deletion mutation in the cardiac myosin-binding protein c gene among japanese.. J Am Coll Cardiol.

[25] Stelzer JE, Fitzsimons DP, Moss RL (2006). Ablation of myosin-binding protein-c accelerates force development in mouse myocardium.. Biophys J.

[26] El-Armouche A, Pohlmann L, Schlossarek S, Starbatty J, Yeh YH (2007). Decreased phosphorylation levels of cardiac myosin-binding protein-c in human and experimental heart failure.. J Mol Cell Cardiol.

[27] Kurada BR, Li LC, Mulherkar N, Subramanian M, Prasad KV (2009). A splice variant of ig20, is indispensable for mapk activation and protection against apoptosis upon tumor necrosis factor-alpha treatment.. J Biol Chem.

[28] Yokoyama T, Vaca L, Rossen RD, Durante W, Hazarika P (1993). Cellular basis for the negative inotropic effects of tumor necrosis factor-alpha in the adult mammalian heart.. J Clin Invest.

[29] Gulick T, Chung MK, Pieper SJ, Lange LG, Schreiner GF (1989). Interleukin 1 and tumor necrosis factor inhibit cardiac myocyte beta-adrenergic responsiveness.. Proc Natl Acad Sci U S A.

