# Supplementary material for: Cardiac Myosin Binding Protein C and MAP-Kinase Activating Death Domain-Containing Gene Polymorphisms and Diastolic Heart Failure
Source: PLoS One. 2012 Apr 17;7(4):e35242. doi: 10.1371/journal.pone.0035242 (PMC3328444; doi:10.1371/journal.pone.0035242)
Supplement: Table S1 — Comparison of MYBPC3 and MADD gene sequence variants in our population, Han Chinese, Yoruba in Ibadan, Nigeria Japanese in Tokyo, Japan and CEPH (Utah residents with ancestry from northern and western Europe). MAF, minor allele frequency; YRI, Yoruba in Ibadan, Nigeria; JPT, Japanese in Tokyo, Japan; CHB, Han Chinese in Beijing, China; CEU, CEPH (Utah residents with ancestry from northern and western Europe). (DOCX) [file pone.0035242.s001.docx]

**Table 1. Comparison of *MYBPC3* and *MADD* gene sequence variants in our population, Han Chinese, Yoruba in Ibadan, Nigeria Japanese in Tokyo, Japan and CEPH (Utah residents with ancestry from northern and western Europe)**

| No. | SNP name | Minor /major allele | MAF of our population | | MAF of CEU | MAF of CHB | MAF of JPT | MAF of YRI |
| --- | --- | --- | --- | --- | --- | --- | --- | --- |
|  |  |  | Case | Control |  |  |  |  |
| 1 | rs3740689 | A/G | 0.463 | 0.423 | 0.700 | 0.295 | 0.511 | 0.067 |
| 2 | rs3824869 | T/C | 0.293 | 0.273 | 0.388 | 0.178 | 0.136 | 0.017 |
| 3 | rs1057233 | C/T | 0.259 | 0.304 | 0.379 | 0.352 | 0.488 | 0.237 |
| 4 | rs2697920 | A/G | 0.463 | 0.480 | 0.342 | 0.433 | 0.356 | 0.875 |
| 5 | rs2856650 | T/C | 0.276 | 0.324 | 0.358 | 0.341 | 0.422 | 0.042 |
| 6 | rs10769253 | A/G | 0.426 | 0.412 | 0.102 | 0.477 | 0.433 | 0.381 |
| 7 | rs2305280 | C/T | 0.398 | 0.372 | 0.814 | 0.514 | 0.444 | 0.566 |
| 9 | rs753992 | C/T | 0.420 | 0.418 | 0.904 | 0.489 | 0.600 | 0.822 |
| 9 | rs7124958 | C/T | 0.406 | 0.381 | 0.902 | 0.489 | 0.466 | 0.570 |
| 10 | rs2290149 | G/A | 0.125 | 0.062 | 0.136 | 0.089 | 0.114 | 0.058 |
| 11 | rs11039179 | T/C | 0.034 | 0.031 | 0.026 | 0.037 | 0.068 | 0 |
| 12 | rs10838692 | T/C | 0.287 | 0.321 | 0.750 | 0.400 | 0.330 | 0.500 |

MAF, minor allele frequency; YRI, Yoruba in Ibadan, Nigeria; JPT, Japanese in Tokyo, Japan; CHB, Han Chinese in Beijing, China; CEU, CEPH (Utah residents with ancestry from northern and western Europe).
